# Optimal dose of cefotaxime in neonates with early-onset sepsis: A developmental pharmacokinetic model-based evaluation

**DOI:** 10.3389/fphar.2022.916253

**Published:** 2022-09-07

**Authors:** Zhen-Hai Shang, Yue-E Wu, Dong-Mei Lv, Wei Zhang, Wen-Qiang Liu, John van den Anker, Yan Xu, Wei Zhao

**Affiliations:** ^1^ Department of Pharmacy, The Affiliated Hospital of Xuzhou Medical University, Xuzhou, China; ^2^ Department of Clinical Pharmacy, Key Laboratory of Chemical Biology (Ministry of Education), School of Pharmaceutical Sciences, Cheeloo College of Medicine, Shandong University, Jinan, China; ^3^ Department of Neonatology, The Affiliated Hospital of Xuzhou Medical University, Xuzhou, China; ^4^ Division of Clinical Pharmacology, Children’s National Hospital, Washington, DC, United States; ^5^ Departments of Pediatrics, Pharmacology & Physiology, Genomics and Precision Medicine, School of Medicine and Health Sciences, George Washington University, Washington, DC, United States; ^6^ Department of Paediatric Pharmacology and Pharmacometrics, University Children’s Hospital Basel, University of Basel, Basel, Switzerland; ^7^ NMPA Key Laboratory for Clinical Research and Evaluation of Innovative Drug, Qilu Hospital of Shandong University, Shandong University, Jinan, China

**Keywords:** cefotaxime, early-onset sepsis, pharmacokinetics analysis, effectiveness, safety

## Abstract

**Objective:** The perspective of real-world study is especially relevant to newborns, enabling dosage regimen optimization and regulatory approval of medications for use in newborns. The aim of the present study was to conduct a pharmacokinetic analysis of cefotaxime and evaluate the dosage used in newborns with early-onset sepsis (EOS) using real-world data in order to support the rational use in the clinical practice.

**Methods:** This prospective, open-label study was performed in newborns with EOS. A developmental pharmacokinetic-pharmacodynamic model of cefotaxime in EOS patients was established based on an opportunistic sampling method. Then, clinical evaluation of cefotaxime was conducted in newborns with EOS using real-world data.

**Results:** A one-compartment model with first-order elimination was developed, using 101 cefotaxime concentrations derived from 51 neonates (30.1–41.3°C weeks postmenstrual age), combining current weight and postnatal age. The pharmacokinetic-pharmacodynamic target was defined as the free cefotaxime concentration above MIC during 70% of the dosing interval (70% fT > MIC), and 100% of neonates receiving the dose of 50 mg/kg, BID attained the target evaluated using the model. Additionally, only two newborns had adverse reactions possibly related to cefotaxime treatment, including diarrhea and feeding intolerance.

**Conclusion:** This prospective real-world study demonstrated that cefotaxime (50 mg/kg, BID) had a favorable efficacy and an accepted safety profile for neonates with EOS.

## Introduction

Early-onset neonatal sepsis (EOS) is a life-threatening systemic infection in newborns with an onset during the first 72 h of life (Hornik et al., 2012; Simonsen et al., 2014). With the improvement of perinatal management and evidence-based application of intrapartum antibiotics, the incidence of EOS has decreased (Polin, 2012). However, EOS is still one of the main causes of morbidity and mortality in neonates, and it presents with a huge challenge because the variable clinical presentation in these infants resulting in a delayed treatment (Scheel and Perkins, 2018).

The selection of empirical antibiotics for treatment of EOS is based on the local epidemiology of the responsible pathogens. In high-income countries, Group B *streptococcus* (GBS) and *Escherichia coli* are the most common pathogens involved in EOS accounting for approximately 70% of infections (Stoll et al., 2011; Schrag et al., 2016; Mukhopadhyay and Puopolo, 2017; Brown and Denison, 2018). Therefore, the initial treatment for EOS is generally ampicillin combined with an aminoglycoside (usually gentamicin) (Polin, 2012). However, a distinctive pathogen distribution exists among neonates with EOS in different countries, for example *Escherichia coli* is the most common pathogens associated with EOS in China (Guo et al., 2019; Jiang et al., 2019). Thus, third-generation cephalosporins, such as cefotaxime, are usually applied to treat EOS in clinical practice, owing to its broad-spectrum antimicrobial activity covering more of the pathogens implicated in neonatal sepsis (Odio, 1995). Cefotaxime undergoes hydrolysis by esterases contained in plasma and the liver to form the active metabolite desacetylcefotaxime, and approximately 50%–60% of a dose is excreted unchanged in the urine, whereas 15%–20% appears as desacetylcefotaxime (Kearns and Young, 1995). The amount of desacetyl cefotaxime formed reduced with increasing liver damage, and cefotaxime clearance markedly declined when the creatinine clearance fell below 10 ml/min (Wise and Wright, 1985; Paap et al., 1991). The dosage adjustment may be necessary for cefotaxime in patients with hepatic and renal dysfunction.

The rational use of cefotaxime in EOS is still hampered by uncertainty about the optimal dose. The dosage regimens of cefotaxime used in different neonatal units vary (75–180 mg/kg/day) due to the absence of a powerful developmental pharmacokinetic-pharmacodynamic study in EOS patients (Leroux et al., 2015b). Pacifici et al. reported that the pharmacokinetics of cefotaxime in newborn babies were primarily studied in the 1980s with a limited number of patients (Pacifici, 2011). The study design and methods limited the power to recommend a precise dosage regimen of cefotaxime in the neonate population using pharmacokinetic data, as the influences of covariates (i.e., hepatic or renal function) on dosage were not fully evaluated. A model-based dosing recommendation was available in children (between the ages of 1 month and 19 years) with Sickle Cell disease. It clearly indicates that the use of a standard dose of cefotaxime is not appropriate for these patients and the dose should be increased in order to optimize efficacy, depending on the children’s clinical presentation and characteristics (Maksoud et al., 2018). The pharmacokinetic profile of most drugs relies on the patient’s covariates and may be influenced by the specific disease. The lack of pharmacokinetic data of the specific population and specific disease may increase the risk of unreasonable use of antibiotics, which could lead to the occurrence of adverse drug reaction or the spread of antibiotic resistance. Thus we aimed to conduct a pharmacokinetic analysis of cefotaxime in EOS patients and evaluate the dosage used in a real-world setting to provide data in terms of pharmacokinetic-pharmacodynamic target achievement, effectiveness and safety to support rational use of cefotaxime in neonates with EOS.

## Methods

### Study design

This prospective, open-label clinical study was conducted in the neonatal intensive care unit (NICU) of the Affiliated Hospital of Xuzhou Medical University, Jiangsu, China. Newborn infants ≤72 h of life who met the standard of starting antibiotic treatment in accordance with NICE guidelines (National Collaborating Centre for and Children’s, 2012) were enrolled in this study, receiving intravenous cefotaxime as part of standard therapy. The standard of initiating antibiotic therapy was as follows: one high risk factor or more than one low risk factor is present (Wu et al., 2021). The exclusion criteria were severe congenital malformation, expected survival time less than the duration of the treatment, undergoing surgery in the first week after birth, participating in another clinical trial, or other circumstances that the investigator deemed unsuitable for enrollment. This study was approved by the Ethics Committee of the Affiliated Hospital of Xuzhou Medical University and abode by the Helsinki Declaration II. Written informed consent was obtained from guardian(s) of each newborn.

### Clinical procedures

Cefotaxime (Huamin Pharmaceutical Co., Ltd., Hebei, China) was administered intravenously within 30 min using a dose of 50 mg/kg/dose BID. The therapeutic effect evaluation was performed by the neonatologist, and the decision to discontinue cefotaxime treatment was made based on clinical manifestations of neonates, the levels of C-reactive protein (CRP) and blood culture results. The first evaluation for discontinuation was made at 36 h after initiating cefotaxime treatment. Cefotaxime would be discontinued if the baby’s clinical presentation associated with sepsis and the levels of CRP remained normal, and blood culture was negative. CRP abnormality was defined as >10 mg/L. If the blood culture was positive, the duration of cefotaxime treatment would last at least 7 days, after which the blood culture required to be reexamined in order to make next decision. After 36 h of cefotaxime treatment, despite negative blood cultures, the baby’s current clinical conditions and the levels of CRP should be reviewed at least once every 24 h, to consider whether it was appropriate to discontinue cefotaxime therapy. Effectiveness and safety profile of cefotaxime were well recorded by clinical research pharmacist during the whole treatment period.

### Sampling and determination of cefotaxime

An opportunistic sampling method was adopted for collecting blood samples (Leroux et al., 2015a). The total number of study-specific blood samples was restricted to two per patient. After routine biochemical examinations, the remaining blood was collected for pharmacokinetic assay. The plasma volume of samples for analyses was 0.1 ml per sample. The time of infusion and sampling was accurately recorded according to standard operating procedure. Each sample was centrifuged for 10 min at 4,000 rpm and 4°C, and plasma samples were stored at −80°C until determination of cefotaxime concentration. High-performance liquid chromatography method with UV detection at 254 nm was adopted to determine cefotaxime plasma concentrations, with tinidazole as internal standard. The chromatographic seperation was performed on a Insustain C18 column (250*4.6 mm, 5 μm, Shimadzu, Japan), with acetonitrile and 0.01 mol/L potassium dihydrogen phosphate solution at a flow rate of 1.0 ml/min. The calibration curve ranged from 0.5 to 200 μg/ml, with 0.5 μg/ml as the lower limit of quantification (LOQ), using 50 μl of plasma samples. The intraday and interday coefficients of variation for the controls were 2.4% and 4.1%, respectively.

### Pharmacokinetic analysis

The non-linear mixed effects modeling program NONMEM V7.4 (Icon Development Solutions, United States) was adopted for pharmacokinetic analysis. The one- and two-compartment model were tested and the results were compared before choosing the structural model. The first-order conditional estimation (FOCE) method with interaction was employed to estimate the pharmacokinetic parameters and their variations. The exponential model was used to estimate the inter-individual differences in pharmacokinetic parameters and expressed as follows:

θi = θ mean*eηi

where *θ* i indicates the value of the ith patient, θmean the typical value of the parameter in the population and ηi the differences in subjects which is supposed to follow a normal distribution with a mean of zero and a variance of ω2.

The forward and backward selection process was employed for covariate analysis. The covariates of birth weight, gestational age, current weight, postnatal age and postmenstrual age were explored as potential variables affecting pharmacokinetic parameters. The impact of each covariate on model parameters was evaluated by the likelihood ratio test.

A covariate was incorporated if the objective function value (OFV) reduction was >3.84 (*p* < 0.05) compared with value of the basic model. All the covariates that had a significant effect were included simultaneously to the model. Then, each covariate was removed in sequence from the model. The covariate was regarded as significant and therefore retained in the final model if the increase in the OFV was more than 6.635 (*p* < 0.01).

Graphical and statistical criteria were adopted to verify the power of the model. Plots of observed concentrations (DV) versus population prediction (PRED), DV versus individual prediction (IPRED), conditional weighted residuals (CWRES) versus time and CWRES versus PRED were applied to verify the performance of the model.

The reliability and stability of the final model was confirmed by a non-parametric bootstrap with resampling and replacement. The non-parametric bootstrap procedure was replicated 1000 times. The values of estimated parameters from the non-parametric bootstrap procedure were compared with those derived from the original data set. PsN (v2.30) was employed to complete the whole procedure in an automated fashion. Eventually, 1000 datasets were simulated with the final population model parameters. R package (v1.2) was used to display QQ-plot and histogram of the NPDE, which was expected to abide by the N (0, 1) distribution. Additionally, pcVPC was used to evaluate the simulation performance.

### Pharmacokinetic-pharmacodynamic target attainment

The pharmacokinetic-pharmacodynamic target was defined as the free cefotaxime concentration above MIC during 70% of the dosing interval (70% fT > MIC). The protein binding rate of cefotaxime was reported to between 27% and 50% (Harding et al., 1981; LeFrock et al., 1982; Patel et al., 1995), and in the cefotaxime label, the protein binding rate is 30%–50%, thus 40% was selected for the calculation of pharmacokinetic-pharmacodynamic target attainment. The MIC of 2 mg/L was assigned as the pharmacokinetic-pharmacodynamic breakpoint for its coverage of most common pathogens (*E. coli* and CoNS) for EOS. The pharmacokinetic-pharmacodynamic target attainment analysis was conducted using the individual empirical Bayesian estimates method by NONMEM software. For each neonate, the simulated drug-free concentration at 70% dosing interval was compared with the MIC value to determine whether the target was reached. The percentage of patients who met the target was calculated. In addition, the AUC_0-24_ at steady-state was calculated by dividing the dose by clearance.

### Effectiveness and safety evaluation

The main indicator for effectiveness assessment of cefotaxime treatment was treatment failure rate. Treatment failure was defined as a recurrence of infection that required extra course of antibiotic therapy within 72 h after ceasing cefotaxime treatment, and/or changing antibiotics owing to exacerbation or no improvement of patient’s conditions, and/or culture-proven pathogens reported resistant to the antibiotic.

The adverse events monitoring covers adverse drug reaction documented in the package insert of cefotaxime and the laboratory testing outliers. The adverse drug reactions recorded in cefotaxime instructions included rash, nausea, vomiting, diarrhea, phlebitis, leukopenia, thrombocytopenia, elevated serum amino transferase level and urea nitrogen and creatinine level. Clinical features and laboratory examination including blood cell analysis, blood gas assay, and biochemical tests were monitored during cefotaxime treatment. Laboratory tests were conducted based on the patient’s condition, not intentionally for study purposes. The causal relation between adverse events and cefotaxime treatment was evaluated by a pediatrician and a clinical pharmacist, and classified as follows: definitely related, probably related, possibly related, not related, or unable to determine.

## Results

### Study population

A total of 54 newborns were enrolled in our study according to the inclusion and exclusion criteria. Three neonates discontinued the trial because of being transferred to another medical center (*n =* 1), change to another antibiotic due to the worsening of patient’s condition before the planned assessment (*n* = 2). Eventually, fifty-one newborns (31 male patients and 20 female patients) accomplished cefotaxime treatment and were included in the following effectiveness and safety evaluation. The clinical baseline characteristics of all neonates were summarized in Table 1. The median (range) values of GA and PNA in the 51 neonates were 35.7 (30.0–41.1) weeks and 1.0 (1.0–3.0) days, respectively. The median (range) values of BW and CW were 2310 (1220–3970) and 2310 (1220–3970) grams respectively.

**TABLE 1:** Baseline characteristics of 51 neonates in effectiveness and safety analysis.

	Median (range)	Number
Patients		51
Male/female		31/20
GA (weeks)	35.7 (30.0–41.1)	
PNA (days)	1.0 (1.0–3.0)	
PMA (weeks)	35.9 (30.1–41.3)	
BW (g)	2310 (1220–3970)	
CW (g)	2310 (1220–3970)	
Commencing antibiotics treatment evaluation		
Patients with one ‘high risk factor’ and ≥0 ‘low risk factor’		8
Patients with 0 ‘high risk factor’ and ≥2 ‘low risk factor’		43
Maternal factors		
Prelabour rupture of membranes		4
Preterm birth following spontaneous labour (before 37 weeks’ gestation)		6
Suspected or confirmed rupture of membranes for more than 18 h in a preterm birth		18
Intrapartum fever higher than 38°C, or confirmed or suspected chorioamnionitis		3
Parenteral antibiotic treatment given to the woman for confirmed or suspected invasive bacterial infection (such as septicaemia) at any time during labour, or in the 24-h periods before and after the birth [This does not refer to intrapartum antibiotic prophylaxis]		1
Clinical indicators		
Altered behaviour or responsiveness		34
Altered muscle tone (for example, floppiness)		2
Feeding difficulties (for example, feed refusal)		3
Feed intolerance, including vomiting, excessive gastric aspirates and abdominal distension		1
Signs of respiratory distress		29
Hypoxia (for example, central cyanosis or reduced oxygen saturation level)		25
Jaundice within 24 h of birth		3
Seizures		1
Need for mechanical ventilation in a preterm baby		15
Need for mechanical ventilation in a term baby		16
Unexplained excessive bleeding, thrombocytopenia, or abnormal coagulation (International Normalised Ratio greater than 2.0)		5
Altered glucose homeostasis (hypoglycaemia or hyperglycaemia)		4
Local signs of infection (for example, affecting the skin or eye)		10

GA, gestational age; PNA, postnatal age; BW, birth weight; CW, current weight.

Eight (15.7%) of the 51 newborns started cefotaxime treatment with one “high risk factor” and ≥0 “low risk factor”, while 43 (84.3%) newborns with 0 “high risk factor” and ≥2 “low risk factor” (Supplementary Material). The major maternal factor for initiating cefotaxime therapy was “suspected or confirmed rupture of membranes for more than 18 h in a preterm birth” in 18 (56.3%) neonates, followed by “preterm birth following spontaneous labor (before 37 weeks’ gestation)” in 6 (11.8%) babies. By contrast, the most common clinical indicator in neonates with EOS was “altered behavior or responsiveness” in 34 (23.0%) newborns, followed by “signs of respiratory distress” in 29 (19.6%) neonates.

### Pharmacokinetic analysis and target attainment

A total of 101 cefotaxime concentrations were available for modeling of population pharmacokinetics. The cefotaxime concentrations ranged from 7.18 to 347.61 μg/ml. Supplementary Figure S1 described the cefotaxime concentration versus time profile. In the current work, the OFV values of the one-compartment model and the two-compartment model were similar, but the one-compartment model with first-order elimination best described the data. The model was parameterized in terms of volume of distribution (V) and clearance (CL) of cefotaxime. An exponential model was adopted to describe inter-individual variability, and residual variability was expressed as a proportional model.

The current weight was included into the basic model using the allometric estimated size approach, which resulted in a significant drop in the OFV of 70.3 points (allometric fixed size approach ΔOFV: 66.3). Postnatal age was confirmed as the most important covariate on CL, with a drop in the OFV of 36.2 units (GA-ΔOFV: 3.62; PMA-ΔOFV: 6.89). No other covariates have a significant effect. The parameter estimates of the final pharmacokinetic model was shown in Table 2. The estimated weight-normalized CL and volume of distribution median (range) values were 0.04 (0.02–0.09) L/h/kg and 0.36 (0.21–0.63) L/kg, respectively. The AUC_0-24_ at steady-state ranged from 974 to 5755 mg*h/L. Clearance of cefotaxime increased allometrically according to current weight.

**TABLE 2:** Population pharmacokinetic parameters of cefotaxime and bootstrap results.

Parameters	Full dataset		Bootstrap		
	Final estimate	RSE (%)	Median		5th–95th
V (L)					
V = *θ*1					
θ1	0.873	5.00	0.872		0.792–0.952
CL (L/h)					
CL = *θ*2×(CW/2310)^θ3^×F_age_					
θ2	0.0803	6.00	0.0802		0.0713–0.0900
θ3	1.68	9.00	1.70		1.42–2.00
F_age_=(PNA/1)^θ4^					
θ4	0.444	15.3	0.452		0.312–0.583
Inter-individual variability (%)					
V	21.1	14.3	20.9		12.8–28.1
CL	20.0	18.9	19.2		13.3–25.3
Residual variability (%)	14.2	15.7	13.7		7.86–17.5

V, volume of distribution; CL, clearance; CW, current weight in gram; PNA, postnatal age in days.

**FIGURE 1:**
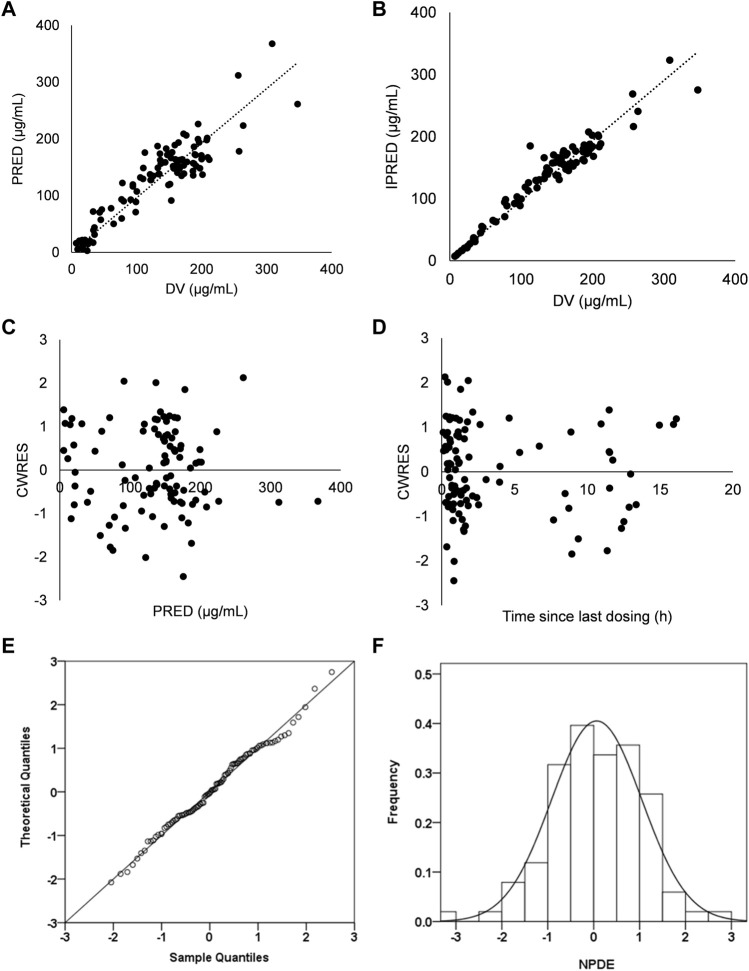
Model evaluation for cefotaxime **(A)** Population predicted concentrations (PRED) versus observed concentrations (DV). **(B)** Individual predicted concentrations (IPRED) versus DV **(C)** Conditional weighted residuals (CWRES) versus PRED. **(D)** CWRES versus time. **(E)** QQ-plot of the distribution of the Normalized Prediction Distribution Errors (NPDE) versus the theoretical N (0,1) distribution. **(F)** Histogram of the distribution of the NPDE.

**FIGURE 2:**
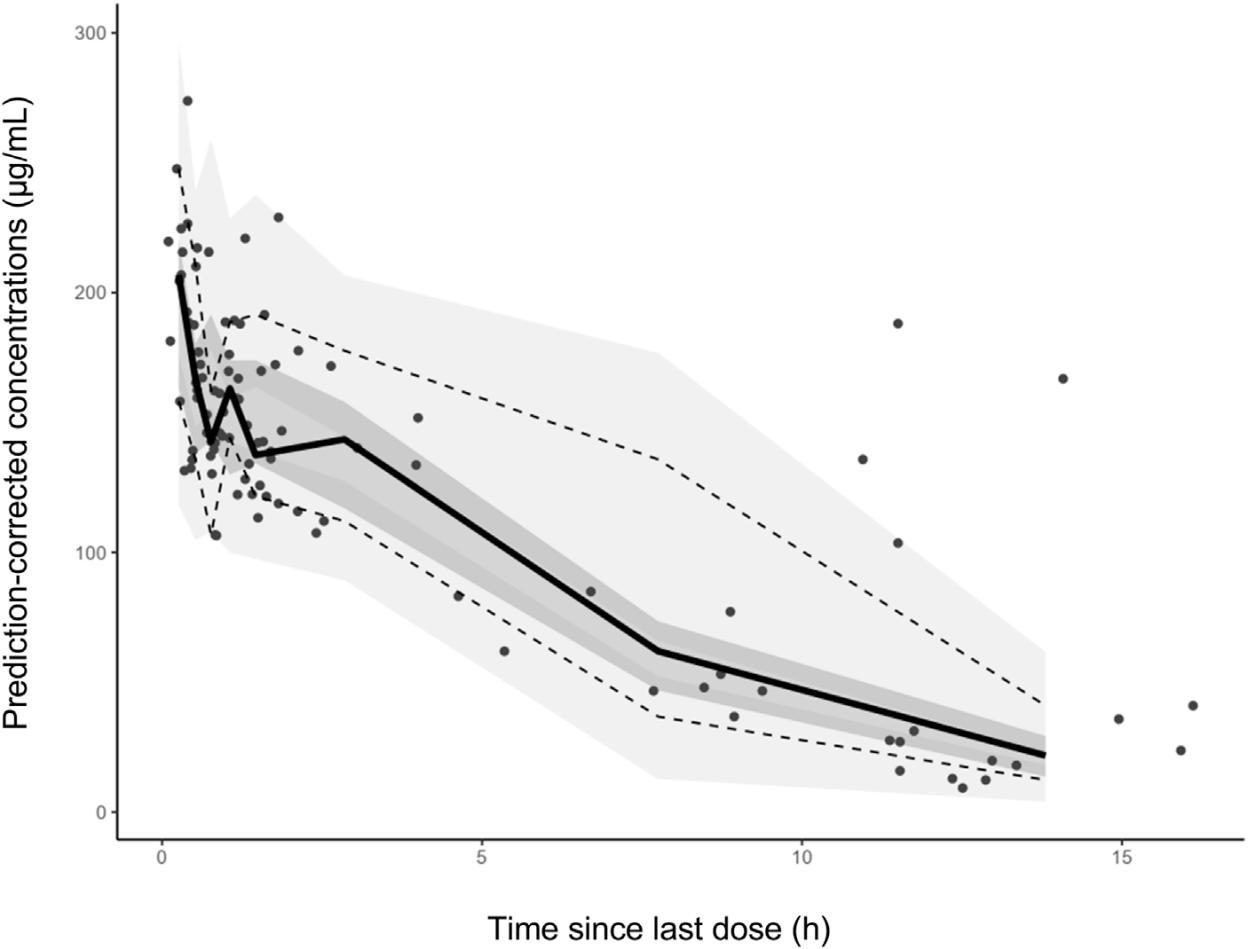
The prediction corrected visual predictive checks. The circles represent the prediction-corrected observed concentrations. The solid line represent the median prediction-corrected observed concentrations and semitransparent gray field represents simulation-based 95% confidence intervals for the median. The observed 5th and 95th percentiles are indicated by dashed lines, and the 95% intervals for the model-predicted percentiles are in a lighter translucent gray.

Favorable goodness-of-fit results for the final model of cefotaxime were validated by means of model diagnostics. As exhibited in Figures 1A,B, the predictions are unbiased. There is no tendency in the diagnostic plots of CWRES versus time and PRED (Figures 1C,D). Additionally, the median parameter estimates deriving from the bootstrap procedure are closely consistent with the values from the final population model, suggesting that the final model is stable and reliable (Table 2). The distribution and histogram of NPDE were in accordance with the theoretical N (0, 1) distribution and density, reflecting a good fit of the model to the individual data (Figures 1E,F). The mean and variance of NPDE were 0.039 and 1, respectively. The pcVPC result is shown in Figure 2. The simulated concentrations are in agreement with the prediction-corrected observed concentrations, validating the predictive performance of the model.

Using this pharmacokinetic model, the dosage regimen of cefotaxime prescribed in the study (50 mg/kg, BID) resulted 100% of neonates achieving the target (70% fT > MIC) at steady state.

### Effectiveness evaluation

As described in Supplementary Table S1, 98.0% (*n* = 50) neonates were successfully cured, and only 2.0% (*n* = 1) newborns experienced treatment failure. The one newborn babies altered antibiotic treatment due to the progression of clinical conditions (switched to meropenem on Day 5). In our study, the median time to start cefotaxime treatment after birth was 3.07 (range 1.0–55.6) hours, while the median duration of cefotaxime therapy in 51 newborns was 6.6 (range 1.5–15.5) days. The median length of hospitalization was 13.0 (range 3.0–36.0) days in all the subjects. Cefotaxime discontinuance criteria were reached in 46 (92%) patients. Approximately half of neonates (23%, 46%) ceased cefotaxime treatment in the third round of discontinuation evaluation (96–144 h), and almost all subjects discontinued cefotaxime therapy in the fourth stage of discontinuation assessment (144–216 h).

### Safety evaluation

In our current study, no subjects ceased cefotaxime treatment or changed the dosage regimen due to AEs. Two AEs occurred in 2 (3.9%) neonates which were regarded as possibly related to cefotaxime therapy, while there were 4 AEs in 4 (7.8%) cases which were recognized as not related to cefotaxime. No patients had AEs which were definitely or probably related to cefotaxime. AEs possibly related to cefotaxime included diarrhea (*n* = 1) and moderate feeding intolerance (*n* = 1). AEs not related to cefotaxime treatment included seizures (*n* = 1) and feeding difficulties (*n* = 3). No infection-related death involved in cefotaxime treatment occurred in the first month after birth.

## Discussion

Our present work using real-world data was the first population pharmacokinetics, effectiveness and safety evaluation of cefotaxime in neonates with EOS in China. The findings of the work revealed that a one-compartment model with first-order elimination best fitted the pharmacokinetics data of cefotaxime. Additionally, this prospective real-world study demonstrated that according to the model results, all of studied neonates treated with cefotaxime (50 mg/kg BID) attained the pharmacokinetic-pharmacodynamic target, which had a favorable efficacy and an accepted safety profile for newborns with EOS.

Since cefotaxime is mainly eliminated *via* a renal route, renal anatomical and function maturation is considered to have an essential influence on cefotaxime CL and dosing in newborn babies. Our results showed that current weight and postnatal age had a crucial impact on cefotaxime clearance, indicating that postnatal renal maturation had an important effect on cefotaxime CL, consistent with principally renally eliminated antibiotics (Rodieux et al., 2015).

Low birth weight is a major risk factor for EOS. In the United States, the overall incidence of EOS was 10.96‰ (10.96 per 1000 live newborns) in very low birth weight (VLBW) babies with a BW of <1500 g, 1.38‰ among low birth weight (LBW) babies with a BW of 1500–2500 g, 0.57‰ among normal birth weight (NBW) babies with a BW > 2500 g (Stoll et al., 2011). In the present study, 28 LBW infants and two VLBW infants were subjected to EOS, accounting for 58.8% of 51 cases. Therefore, for LBW or VLBW infants, once suspected or confirmed EOS, prompt and effective treatment should be adopted. Additionally, another important risk factor for neonatal sepsis is maternal membrane rupture. An obstetric risk factor—membrane rupture >18 hours—was found in 13.5% of group B streptococcal (GBS) cases and 26.7% of other sepsis (Schuchat et al., 2000). Compared to neonatal clinical indicators, maternal risk factors are more important for the diagnosis of EOS and the starting of antibiotic treatment. In our study, the top three reasons to initiate antibiotic therapy were maternal risk factors, including “suspected or confirmed rupture of membranes for more than 18 h in a preterm birth”, “preterm birth following spontaneous labour (before 37 weeks’ gestation)”, and “prelabour rupture of membranes”.

Antibiotics are frequently prescribed in the treatment of neonatal sepsis due to the high rates of incidence and mortality (Hornik et al., 2012; Oliver et al., 2017; Stocker et al., 2017). However, many medications used in neonatal clinical practice are unlicensed, owing to the absence of evidence-based dosing regimen (Schrier et al., 2020). Undoubtedly, empirical antibiotic treatment in newborns brings the risk of either drug-resistant bacteria due to underdose or side effects owing to overdose. The adoption of real-world evidence (RWE) is becoming increasingly essential for the evaluation of effectiveness and safety and the rational use of drugs in children (Lamberti et al., 2018; Lasky et al., 2020). A combination of ampicillin and an aminoglycoside was recognized as a standard regimen of EOS. However, aminoglycosides are not allowed to treat EOS in China because of a higher risk of ototoxicity. Instead, local neonatologists are more likely to prescribe third-generation cephalosporins, such as cefotaxime, based on its antibacterial spectrum and the distribution of pathogens. Obviously, it is crucial to generate real world evidence derived from real world data of alternative medication for neonatal EOS.

In the effectiveness and safety evaluation, in view of ambiguous signs and symptoms, low detection rate of blood culture and requirement for timely diagnosis and treatment, we designed the clinical trial according to NICE guidelines including maternal factors or clinical indicators, to assess the effectiveness and safety of cefotaxime in newborn babies with EOS. In the present study, the average time to initiate cefotaxime therapy after birth was 3.07 h, longer than 2.0 h in previous research (Stocker et al., 2017), which may be explained by the discrepancy of clinical practice in different medical centers. In addition, the average duration of cefotaxime treatment was 6.6 days, longer than 5 days in Cordero’s study (Cordero and Ayers, 2003). This may be partially explained by the fact that delayed start of antibiotics treatment resulted in prolonged administration of antimicrobial agents (>5 days). Extended treatment duration of empirical antibiotics, especially third-generation cephalosporins, is associated with subsequent adverse outcomes, including late onset sepsis (LOS), necrotizing enterocolitis (NEC), invasive candidiasis and death (Cotten et al., 2009; Alexander et al., 2011; Kuppala et al., 2011; Cantey et al., 2018; Raba et al., 2019). Therefore, considering severe harmful outcomes implicated in prolonged use of antibiotics, the need to develop a precise strategy which can determine the duration of antibiotics treatment is essential for lower incidence of severe adverse outcomes.

As for effectiveness evaluation, cefotaxime demonstrated favorable therapeutic effect in the treatment of EOS. In the current work, 98.0% (*n* = 50) neonates with EOS were effectively cured, and only 2.0% (*n* = 1) newborns were evaluated as treatment failure. There is no definite value (27%–50%) for the protein binding rate of cefotaxime in human according to the reported data (Harding et al., 1981; LeFrock et al., 1982; Patel et al., 1995). In the cefotaxime label, the protein binding rate is 30%–50%, thus a median value of 40% was selected for PD target evaluation. The pharmacokinetic-pharmacodynamic target attainment was reached in 51 (100%) neonates, which enabled a consistent conclusion, reflecting a satisfying dosing regimen for this population. From the perspective of safety, there were limited adverse events implicated in cefotaxime treatment in newborns (Kearns and Young, 1995). The side effects are mainly presented as hypersensitivity and gastrointestinal reactions, and cefotaxime rarely leads to nephrotoxicity and seizures (Roberts et al., 2014; Fanos and Dall'Agnola, 1999). The acceptable safety profile found in our study is consistent with these findings. Despite some studies have noted a rise in the incidence of invasive candidiasis, necrotizing enterocolitis and late onset sepsis because of the initial use of cefotaxime (Bryan et al., 1985; Manzoni et al., 2006; Polin, 2012), these serious adverse outcomes did not occur in our present study. However, out of prudence and safety, cefotaxime would be ceased promptly if the baby’s clinical presentation associated with sepsis and the levels of CRP turned normal after evaluation, and meanwhile these cases should be paid more attention and given careful nursing during cefotaxime treatment, in order to avoid the severe adverse outcomes involved in cefotaxime.

Several limitations existed in our study. Extremely low birth weight (<1000 g) neonates are missing in the present study. Thus, our results can simply not be extrapolated to this population. Additionally, cefotaxime performed well in the effectiveness and safety evaluation over a one-month follow-up period. The long-term safety of cefotaxime in larger samples needs to further investigation.

## Conclusion

We evaluated the effectiveness and safety of cefotaxime using real-world data, and the drug exhibited a favorable clinical benefits and safety in neonates. Innovative approach should be promoted to assess off-label drugs in newborns for rational use.

## Data Availability

The original contributions presented in the study are included in the article/Supplementary Material, further inquiries can be directed to the corresponding authors.
