# Supplementary material for: Optimal dose of cefotaxime in neonates with early-onset sepsis: A developmental pharmacokinetic model-based evaluation
Source: Front Pharmacol. 2022 Sep 7;13:916253. doi: 10.3389/fphar.2022.916253 (PMC9490083; doi:10.3389/fphar.2022.916253)
Supplement: Supplementary file 1 [file DataSheet1.docx]

**High risk factors for EOS**

**Maternal factors:** Parenteral antibiotic treatment given to the woman for confirmed or suspected invasive bacterial infection (such as septicaemia) at any time during labour, or in the 24-hour periods before and after the birth [This does not refer to intrapartum antibiotic prophylaxis]; suspected or confirmed infection in another baby in the case of a multiple pregnancy.

**Clinical indicatoers of newborns:** Respiratory distress starting more than 4 hours after birth; seizures; need for mechanical ventilation in a term baby; signs of shock.

**Low risk factors for EOS**

**Maternal factors:** Invasive group B streptococcal infection in a previous baby; maternal group B streptococcal colonisation, bacteriuria or infection in the current pregnancy; prelabour rupture of membranes; preterm birth following spontaneous labour (before 37 weeks' gestation); suspected or confirmed rupture of membranes for more than 18 hours in a preterm birth; intrapartum fever higher than 38°C, or confirmed or suspected chorioamnionitis.

**Clinical indicatoers of newborns:** Altered behaviour or responsiveness; altered muscle tone (for example, floppiness); feeding difficulties (for example, feed refusal); feed intolerance, including vomiting, excessive gastric aspirates and abdominal distension; abnormal heart rate (bradycardia or tachycardia); signs of respiratory distress; hypoxia (for example, central cyanosis or reduced oxygen saturation level); jaundice within 24 hours of birth; apnoea; signs of neonatal encephalopathy; need for cardio–pulmonary resuscitation; need for mechanical ventilation in a preterm baby; persistent fetal circulation (persistent pulmonary hypertension); temperature abnormality (lower than 36°C or higher than 38°C) unexplained by environmental factors; unexplained excessive bleeding, thrombocytopenia, or abnormal coagulation (International Normalised Ratio greater than 2.0); oliguria persisting beyond 24 hours after birth; altered glucose homeostasis (hypoglycaemia or hyperglycaemia); metabolic acidosis (base deficit of 10 mmol/litre or greater); local signs of infection (for example, affecting the skin or eye).

**Table Legends**

**Supplementary Table 1.** Effectiveness and safety outcome measures.

**Figure Legends**

**Supplementary Figure 1.** Cefotaxime concentration versus time since last dosing. DV: detected values of cefotaxime concentrations.

**Supplementary Table 1.** Effectiveness and safety outcome measures.

|  | Patients （n=51） |
| --- | --- |
| **Cefotaxime treatment** |  |
| Time to begin cefotaxime therapy after birth (h) | 3.07 (1.0 - 55.6) |
| Duration of cefotaxime treatment (days) | 6.6 (1.5 - 15.5) |
| Cefotaxime discontinuation evaluation |  |
| Stage 1 (36-48h) (%) | 4 (8%) |
| Stage 2 (48-96h) (%) | 15 (30%) |
| Stage 3 (96-144h) (%) | 23 (46%) |
| Stage 4 (144-216h) (%) | 46 (92%) |
| **Length of hospitalization (days)** | 13.0 (3.0 - 36.0) |
| **Treatment failure (%)** | 1 (2.0%) |
| 1) Recurrence of infection that required additional courses of antibiotic therapy within 72 h after ending the initial course of cefotaxime treatment | 0 |
| 2) Changing antibiotics because of no improvement or deterioration | 1 |
| 3) Blood culture isolate reported resistant to the antibiotic | 0 |
| **Pharmacokinetic-pharmacodynamic target attainment** | 100% |
| **Mortality (%)** |  |
| 1-month mortality^#^ | 0 (0%) |
| **Adverse events** |  |
| Definitely related | 0 (0%) |
| Probably related | 0 (0%) |
| Possibly related | 2 (3.9%) |
| Not related | 4 (7.8%) |
| Unable to determine | 0 (0%) |

Data are presented as median (range), n (%). **^#^** Infection-related death in the first month of life accompanied by cefotaxime treatment.

**
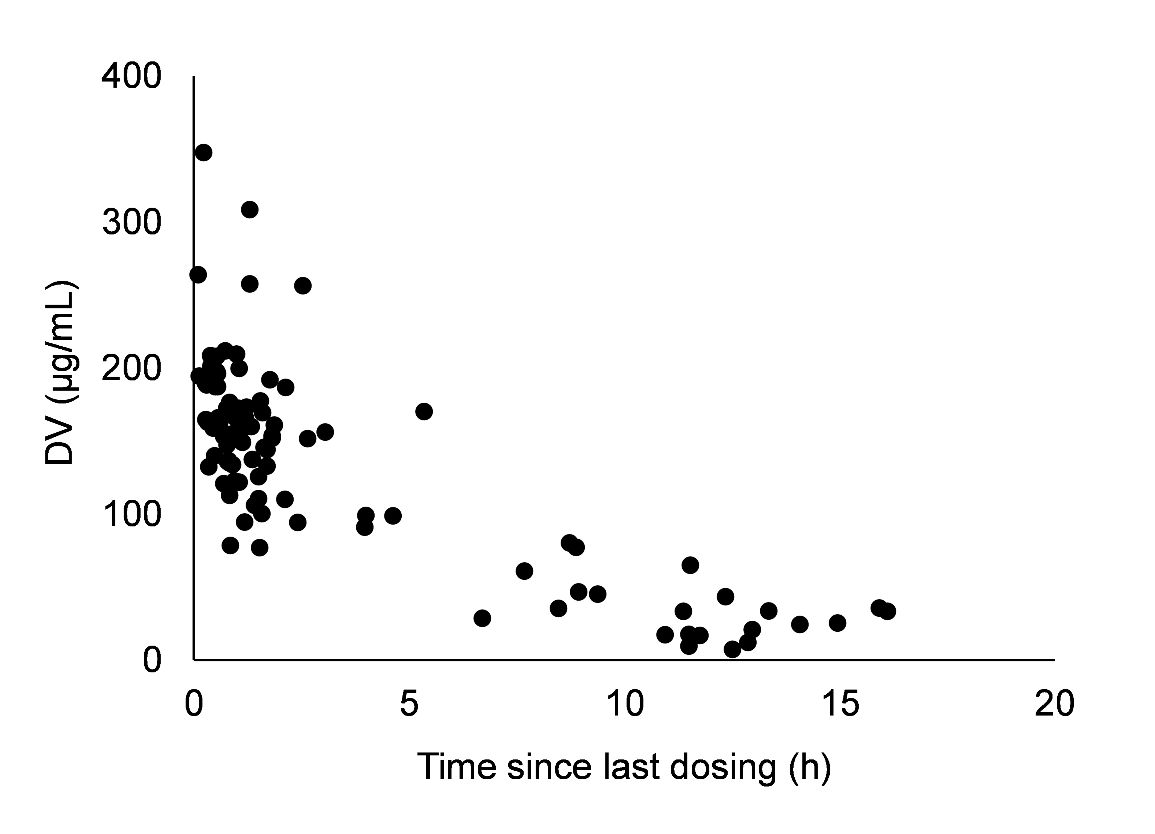
**

**Supplementary Figure 1.** Cefotaxime concentration versus time since last dosing. DV: detected values of cefotaxime concentrations.
